# Synthesis and Phase Behavior of Poly(*N*-isopropylacrylamide)-b-Poly(L-Lysine Hydrochloride) and Poly(*N*-Isopropylacrylamide-co-Acrylamide)-b-Poly(L-Lysine Hydrochloride)

**DOI:** 10.3390/ma7075305

**Published:** 2014-07-22

**Authors:** Milica Spasojević, Joop Vorenkamp, Mark R. P. A. C. S. Jansen, Paul de Vos, Arend Jan Schouten

**Affiliations:** 1 Department of Polymer Chemistry, Zernike Institute for Advanced Materials, University of Groningen, Nijenborgh 4, 9747 AG Groningen, The Netherlands; E-Mails: m.spasojevic@umcg.nl (M.S.); e.j.vorenkamp@rug.nl (J.V.); m.r.p.a.c.s.jansen@rug.nl (M.R.P.A.C.S.J.); a.j.schouten@rug.nl (A.J.S.); 2 Department of Pathology and Medical Biology, Section of Immunoendocrinology, University Medical Center Groningen, University of Groningen, Hanzeplein 1, EA11, 9700 RB Groningen, The Netherlands

**Keywords:** diblock copolymers, ATRP, ROP, thermal behavior

## Abstract

The synthesis of poly(*N*-isopropylacrylamide)-b-poly(L-lysine) and poly(*N*-isopropylacrylamide-co-acrylamide)-b-poly(L-lysine) copolymers was accomplished by combining atom transfer radical polymerization (ATRP) and ring opening polymerization (ROP). For this purpose, a di-functional initiator with protected amino group was successfully synthetized. The ATRP of *N*-isopropylacrylamide yielded narrowly dispersed polymers with consistent high yields (~80%). Lower yields (~50%) were observed when narrowly dispersed random copolymers of *N*-isopropylacrylamide and acrylamide where synthesized. Amino-terminated poly(*N*-isopropylacrylamide) and poly(*N*-isopropylacrylamide-co-acrylamide) were successfully used as macroinitiators for ROP of *N*_6_-carbobenzoxy-L-lysine *N*-carboxyanhydride. The thermal behavior of the homopolymers and copolymers in aqueous solutions was studied by turbidimetry, dynamic light scattering (DLS) and proton nuclear magnetic resonance spectroscopy (^1^H-NMR).

## 1. Introduction

Poly(*N*-isopropylacrylamide) (PNIPAAm) is one of the most intensively studied synthetic polymers for use in controlled drug delivery [1,2,3], cell-sheet engineering [4,5,6,7], as a biosensor [8,9,10] or in tissue engineering [11,12,13]. PNIPAAm polymer brushes with a low polydispersity showed to be resistant towards protein adsorption [14,15]. The most interesting feature of poly(*N*-isopropylacrylamide) is the reverse solubility upon heating in water. This thermo-responsive behavior originates from the ability of the polymer to undergo a change from a dissolved coil to a collapsed globule when the temperature is raised above 32 °C. This transition temperature is known as the lower critical solution temperature (LCST). It is assumed that below the LCST, water-swollen polymer brushes repel proteins whereas collapsed, hydrophobic polymer brushes above the LCST adsorb proteins. However, recent reports have shown that PNIPAAm polymer brushes do not always collapse above the LCST and proteins do not adsorb to all PNIPAAm coatings or hydrogels above the LCST [5,6,7,14,15]. The LCST can be shifted by copolymerizing PNIPAAm with other more hydrophilic or hydrophobic monomers [2,16,17,18,19,20,21].

NIPAAm can be polymerized in various ways. High molecular weight (*M*_w_) PNIPAAm was obtained via radical polymerization. Polymers produced in this way have the disadvantage of a high polydispersity index (PDI). Narrowly dispersed PNIPAAms with the desired molecular weights can be obtained in a controlled manner via nitroxide mediated polymerization (NMP) [22], anionic polymerization [23,24], reversible addition–fragmentation chain transfer (RAFT) [25,26,27,28] and atom transfer radical polymerization (ATRP) [29,30,31,32,33].

Initially, ATRP was considered inappropriate for controlled polymerization of NIPAAm [30]. However, side reactions that disturbed the activation-growth-deactivation process in the ATRP of NIPAAm have been avoided by careful selection of the starting materials such as solvent, initiator and ligand. Polar solvents, chlorinated initiators and a strong ligand such as tris(2-dimethylaminoethyl) amine (Me_6_TREN) have been shown to be crucial in suppressing the deactivation of the catalyst caused by competitive coordination of (P)NIPAAm, displacement of the terminal halide atom by the amide group and disproportionation of the copper catalyst [30,32,33].

Besides a good control over molecular weight, polydispersity, composition and architecture ATRP enables a facile tailoring of end-group functionality. In a proper ATRP, the end groups of the prepared polymer chains originate from the initiator. Therefore, the end-functionality of the polymer chains is determined by the functionality of the initiator. Polymer chains with certain end groups can be used further on as macroinitiators, such as amino or alcohol end-groups that can initiate ring opening polymerizations and thus facilitate the copolymer synthesis.

The most frequently used method for synthetizing polypeptides is ring opening polymerization of α-amino acid-*N*-carboxyanhydrides (NCAs). Ring opening polymerization is a straightforward method, which allows for the synthesis of polypeptides with high molecular weights in good yields and large quantities. Besides amines, ROP can be initiated by alcohols [34,35], water and metal salts [36,37], transition-metal amine complexes [38,39] and amine salts [33,40,41]. Hybrid block copolymers containing synthetic and natural polymer blocks can be prepared by combining ROP of NCAs and some other polymerization techniques such as ATRP [33,42], RAFT [43], NMP [43,44] and anionic polymerization [45]. The synthesis of these block copolymers can be performed through two different routes. The first route employs an amino-terminated synthetic polymer, which can initiate the ROP of NCAs producing the peptide block. Alternatively, a halide-terminated polypeptide can act as an initiator in preparation of synthetic polymer blocks via ATRP.

In this paper, the synthesis of PNIPAAm-b-PLL diblock copolymers by combining ATRP and ROP will be described. For this purpose, a di-functional initiator was prepared. Diblock copolymers were successfully synthetized when the di-functional initiator was used first to polymerize NIPAAm in a controlled manner via ATRP and subsequently amino-functionalized PNIPAAm initiated ROP of L-lysine-NCA. The molecular weights of the synthetized polymers were determined using GPC, ^1^H-NMR as described in the Supplementary Data and from the conversion. Due to the thermo-responsive nature of PNIPAAm blocks, it is difficult to predict whether these diblock copolymers will provide improved surface properties of the alginate-based capsules used for the cell encapsulation or whether they will cause more issues such as protein adsorption followed by the cell adhesion and graft failure. Therefore, we decided to shift the LCST of PNIPAAm towards higher values (above 37 °C) by copolymerizing NIPAAm with acrylamide as a comonomer. Random copolymerization of N-isopropylacrylamide and acrylamide has been reported in literature [20,43]. It has been shown that the incorporation of acrylamide (20%) in a PNIPAAm chain increases the LCST by approximately 10 °C [20,46,47]. A random copolymer of NIPAAm and AAm was successfully prepared by ATRP and the amino-terminated copolymer was used as a macroinitiator in the ROP of L-lysine-NCA.

## 2. Results

The PNIPAAm-b-PLL diblock copolymer can be prepared by combining atom transfer radical polymerization and ring opening polymerization as described in Scheme I. The same synthesis route can be used for the preparation of the PNIPAAm-PAAm-b-PLL copolymer where the AAm monomer is copolymerized with NIPAAm via ATRP to give a random copolymer PNIPAAm-AAm in the first synthesis step.

**Scheme I:**
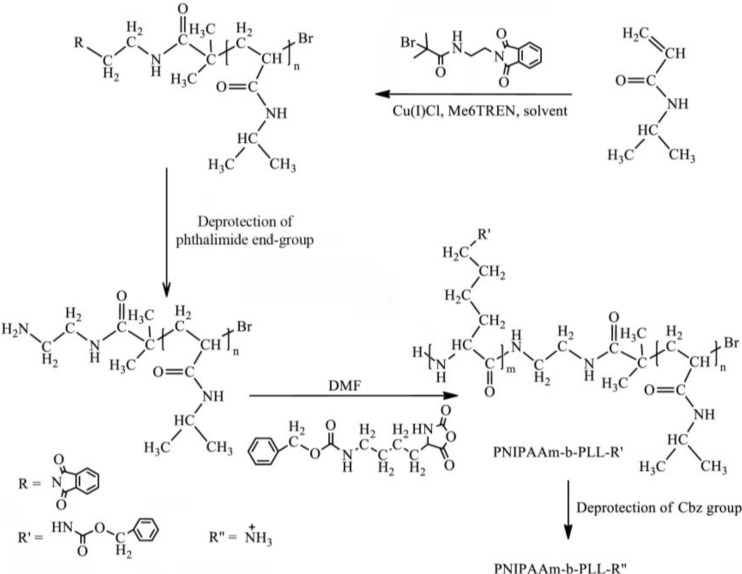
Synthesis route of PNIPAAm-b-PLL.

### 2.1. ATRP of N-Isopropylacrylamide

**Figure 1:**
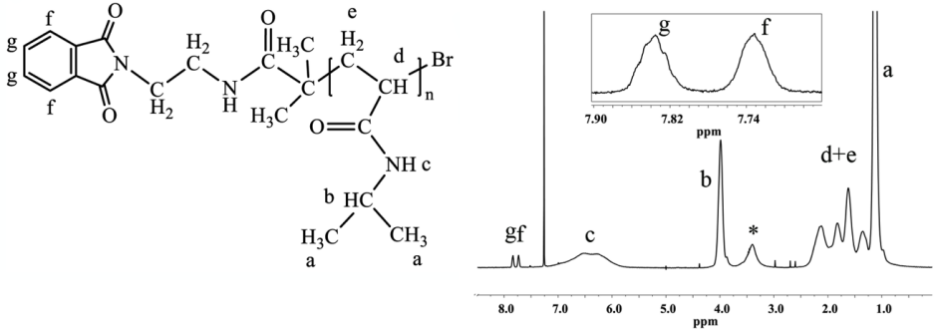
^1^H-NMR spectrum of PNIPAAm prepared with phthalimide protected initiator with magnification of the end-group area. The end-group protons give multiplets isolated from peaks of the polymer backbone. The spectrum was recorded in CDCl_3_, 64 scans and 12 s relaxation delay (determined by T1 test), (*, water).

Because of the mechanism of ATRP, the end-group functionality of the initiator is the same as that of the final polymer, which allows for the straightforward calculation of the degree of polymerization and molecular weight from end-group analysis by ^1^H-NMR. The multiplets at 7.73 and 7.84 ppm correspond to four protons of the phthalimide end group (Figure 1). The ratio between the integrated area under the peak at 3.99 ppm, which corresponds to one proton of PNIPAAm (–CH–(CH_3_)_2_), and one quarter of the integrated area of the multiplets at 7.73 and 7.84 ppm, defines the degree of polymerization of PNIPAAm (Figure 1).

Determination of the molecular weight via end group analysis can be performed up to molecular weights of ~20 kg/mol. Beyond 20 kg/mol, the end group analysis becomes unreliable.

The polymers were also analyzed by gel permeation chromatography (GPC). From this analysis, it became apparent that there was a consistently large difference between the theoretical molecular weight (based on conversion) and the molecular weight determined by GPC. Molecular weights determined by universal calibration were 2–3 times higher than the theoretical molecular weights. When light scattering was used as a detector the difference was somewhat lower (50%–100%). The molecular weight determined by ^1^H-NMR was comparable to the theoretical values. The molecular weights of PNIPAAm homopolymers determined with different methods are presented in Table 1.

**Table 1:** Comparison between theoretical and molecular weights of PNIPAAm found by end-group analysis, GPC light scattering (LS) and GPC universal calibration (UC).

Ratio M:I:C:L	Conversion (^1^H-NMR), %	Theoretical M_n_, kg/mol	^1^H-NMR M_n_, kg/mol	GPC			
				LS		UC	
				M_n_, kg/mol	PDI	M_n_, kg/mol	PDI
50:1:2:2	92.5	5.20	6.60	9.90	1.11	15.90	1.05
100:1:2:2	90.4	10.20	13.30	16.70	1.16	20.30	1.13
200:1:2:2	82.5	18.70	18.60	27.20	1.24	35.80	1.10

M:I:C:L = monomer:initiator:copper:ligand.

All PNIPAAm polymers were narrowly dispersed. PDI values of these polymers were ≤1.2.

### 2.2. Random Copolymerization of N-Isopropylacrylamide and Acrylamide by ATRP

PNIPAAm is a thermo-responsive polymer. Below the low critical solution temperature (LCST = 32 °C) PNIPAAm is water soluble, but at higher temperatures (>32 °C) a transition from a dissolved coil to a collapsed globule occurs and PNIPAAm becomes insoluble in water. The LCST of PNIPAAm can be tailored by incorporation of either hydrophilic or hydrophobic monomers in the PNIPAAm chain [2,16,17,18,19,20,21]. 

In order to shift the LCST of PNIPAAm towards temperatures above 37 °C (normal human body temperature), random copolymerization of NIPAAm and AAm by ATRP was performed. ^1^H-NMR analysis of the reaction mixture after 24 h has shown similar conversions of both monomers, which indicated successful copolymer formation, confirmed by the GPC chromatogram of the copolymers. However, the conversions were lower than the conversions achieved when only NIPAAm was polymerized under the same conditions (~40% *versus* 90%). In order to increase the incorporation of monomer units into the polymer chain, different polymerization conditions were examined.

Four different solvent systems were tested: DMSO, DMF, DMF/water and isopropanol. In isopropanol the conversion cannot be determined via ^1^H-NMR due to overlapping of polymer/monomer peaks with peaks coming from the solvent. Therefore, the solvent selection was based on the polymer yield. Although the DMF/water (v/v = 75/25) solvent system provided the highest yield (50%), the PDI value of obtained polymer was higher than in other solvent systems (1.54 *versus* 1.37 in isopropanol, 1.38 in DMSO and 1.19 in DMF). Comparable yields (~40%) were obtained when DMSO and DMF were used as solvent, whereas in isopropanol, 10% less polymer was obtained. Therefore, DMF has been chosen as the most appropriate solvent from the tested solvent systems.

The influence of reaction time and temperature was also investigated. Longer reaction times (>1 day) did not improve the conversion of monomers into copolymer. Elevated temperatures (60 and 110 °C) even caused a contrary effect. Lower conversions were observed when polymerizations were performed at temperatures above room temperature (20% at 60 °C and 13% at 110 °C).

The composition of PNIPAAm-PAAm copolymer was tuned by varying the NIPAAm/AAm ratio in the reaction mixture. It has been shown that this ratio also has an influence on the monomer conversion. By increasing the AAm monomer concentration from 20 to 30 mol%, the final conversion decreases from 43% to 38%. 

The improvement in conversion was accomplished when the initial concentration of monomers in reaction mixture was increased from 33 to 50 wt%. The increase in monomer concentration did not interfere with controlled nature of ATRP and copolymers with a low dispersity were obtained. The PNIPAAm-PAAm copolymers successfully prepared by ATRP are listed in Table 2. The molecular weights obtained by GPC are higher than values calculated from ^1^H-NMR. 

**Table 2:** Random copolymers of NIPAAm and AAm prepared by ATRP (atom transfer radical polymerization) with the phthalimide initiator in DMF for 24 h at room temperature.

Entry	M1% M2%	TIMC, % M:I:C:L	Conversion, % (^1^H-NMR)	^1^H-NMR M_n_, kg/mol	GPC			
					LS		UC	
					M_n_, kg/mol	PDI	M_n_, kg/mol	PDI
1	80, 20	33, 250:1:4:4	38.1	-	14.60	1.19	19.00	1.15
2	80, 20	50, 250:1:4:4	42.9	-	17.00	1.25	20.20	1.21
3	70, 30	50, 250:1:4:4	37.5	10.35	14.90	1.45	20.90	1.23
4	80, 20	50, 250:1:2:2	53	13.43	18.00	1.25	24.60	1.10
5	80, 20	50, 500:1:2:2	50.8	27.85	40.00	1.38	40.90	1.26

M1, N-isopropylacrylamide; M2, acrylamide; TIMC, total initial monomer concentration; M:I:C:L = monomer:initiator:copper:ligand. First two samples were not analysed by ^1^H-NMR.

### 2.3. Deprotection of the Phthalimide Protecting Group

Before either PNIPAAm or PNIPAAm-PAAm can be used as a macroinitiator in the ring opening polymerization of L-lysine-NCA, the amino group has to be deprotected by removing of the phthalimide end group.

**Figure 2:**
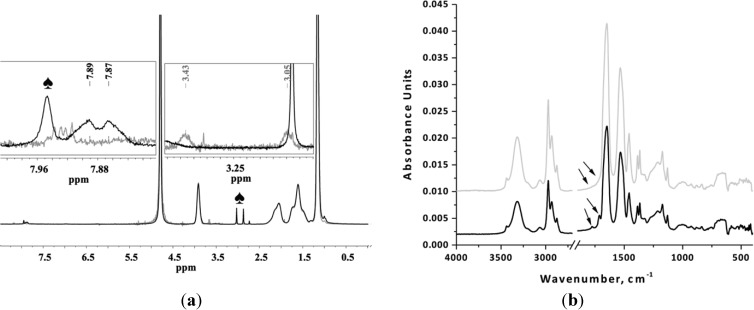
(**a**) ^1^H-NMR and (**b**) FTIR spectra of PNIPAAm before (black) and after (grey) removal of phthalimide-protecting group. ^1^H-NMR spectra were recorded in D_2_O; number of scans ~ 32, relaxation delay 12 s (♠, residues of DMF).

The removal of phthalimide end-group was confirmed by the absence of peaks at 7.89 and 7.87 ppm (aromatic protons of phthalimide group) and by the appearance of the peaks at 3.43 and 3.05 ppm (two CH_2_ groups adjacent to the newly formed amine end-group) (Figure 2a). FTIR analysis shows the absence of bands from the stretching vibrations of phthalimide C=O groups (1715 cm^−1^ and 1774 cm^−1^) after the deprotection step (Figure 2b).

### 2.4. ROP of Lysine-NCA

Before amino-terminated PNIPAAm and PNIPAAm-PAAm were used as macroinitiators in the ROP, L-lysine-NCA was first successfully synthetized. The purity of the lysine monomer was confirmed by melting point determination and ^1^H-NMR. The melting point of the obtained material was ~100 °C and is in good agreement with the values reported in literature [48,49].

**Figure 3:**
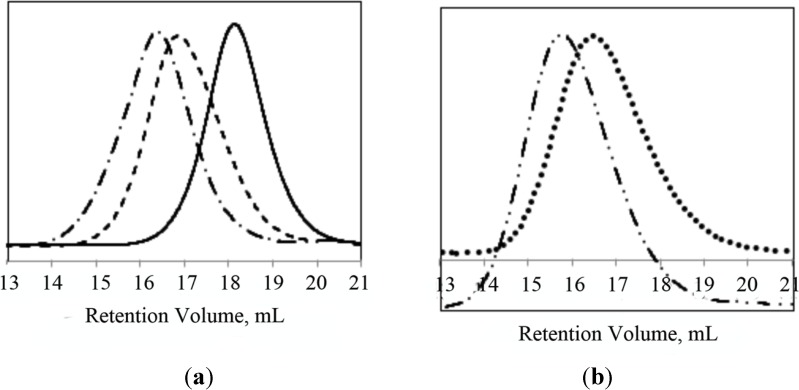
GPC chromatograms of (**a**) PNIPAAm_58_ macroinitiator (─), PNIPAAm_58_-b-PLL_44_ (─ ─), PNIPAAm_58_-b-PLL_103_ (─ • ─); (**b**) PNIPAAm_102_-PAAm_26_ (• • • •) and PNIPAAm_102_-PAAm_26_-b-PLL_90_ (─ • • ─).

PNIPAAm-b-PLL and PNIPAAm-PAAm-b-PLL copolymers were characterized by GPC and ^1^H-NMR. The molecular weights of the synthetized block copolymers were determined by GPC with PMMA standards in DMF as an eluent. Successful diblock copolymer formation was confirmed by the shift to higher molecular weights in the GPC chromatogram of the diblock copolymers (PNIPAAm-b-PLL or PNIPAAm-PAAm-b-PLL) compared to their corresponding macroinitiators (PNIPAAm or PNIPAAm-PAAm). GPC chromatograms of PNIPAAm/PNIPAAm-PAAm macroinitiators and their diblock copolymers are presented in Figure 3.

**Figure 4:**
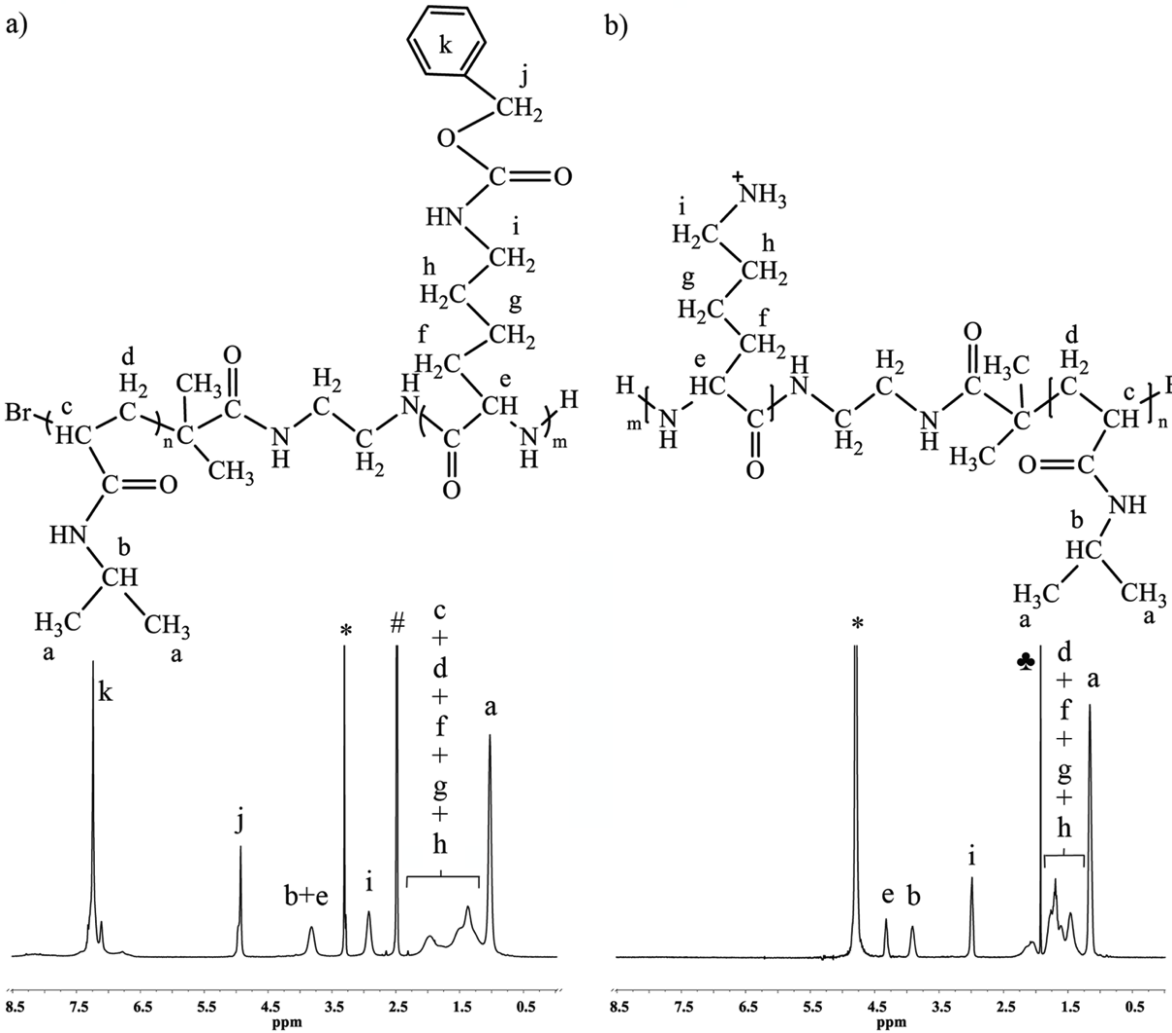
^1^H-NMR spectra of PLL (**a**) before and (**b**) after treatment with HBr in acetic acid. The spectra were recorded with relaxation delay of 12 s, 32 scans in DMSO-d6 (**a**) and D_2_O (**b**), # DMSO-d6; * water; ♣ residues of acetic acid.

Alongside the confirmation of the diblock copolymer structure, ^1^H-NMR was also used to determine the degree of polymerization and therefore, the molecular weight of the PLL block (Table 3) as well as the molecular weight of the entire copolymer. The degree of polymerization was determined from the peak at 2.93 ppm (CH_2_–NHCOO) of PLL and the peak area from 2.25 to 0.8 ppm which corresponds to nine protons (CH(CH_3_)_2_, BrCHCH_2_ and BrCHCH_2_) from PNIPAAm and six protons (CH–(CH_2_)_3_–CH_2_–NH) from PLL, as described in the Supplementary data (see Figure 4a). Due to overlapping of L-lysine-NCA peaks and PLL peaks, the conversions cannot be determined from ^1^H-NMR of the reaction mixture. Therefore, the theoretical molecular weight of PLL block was calculated from the yield. However, this method is potentially not very accurate due to losses incurred during the purification procedure. This also explains differences in theoretical M_n_ and molecular weights calculated from ^1^H-NMR. The molecular weights of the PLL block calculated from the yield as well as those determined via ^1^H-NMR analysis, and the molecular weights and PDI of the diblock copolymers determined by GPC are presented in Table 3.

In order to prepare NH_3_^+^ side groups in the PLL block, required for linkage with alginate through the polyelectrolyte complex, the carbobenzyloxy (Cbz) group of the PLL has to be removed. This was achieved by treating the polymer with concentrated strong acid solution as described in the Materials and Methods section. The complete removal of the Cbz-group was confirmed by the absence of peaks at 7.24 (–O–CH_2_–C_6_H_5_) and 4.93 ppm (–O–CH_2_–C_6_H_5_) (Figure 4b).

**Table 3:** Diblock copolymers of PNIPAAm/PNIPAAm-PAAm and PLL were characterized by GPC and ^1^H-NMR. M_n_ of the PLL blocks was calculated from yields as well as from ^1^H-NMR.

Sample	Yield, %	M_n_(PLL), kg/mol		GPC			
		From yields	By ^1^H-NMR	LS		UC	
				M_n_, kg/mol	PDI	M_n_, kg/mol	PDI
PNIPAAm_58_-b-PLL_44_	72.6	10.90	11.50	26.70	1.22	26.40	1.22
PNIPAAm_58_-b-PLL_103_	79.4	23.80	27.00	37.90	1.18	38.00	1.18
PNIPAAm_118_-b-PLL_54_	73.4	11.00	14.20	37.50	1.18	37.40	1.18
PNIPAAm_118_-b-PLL_101_	95.0	28.50	26.50	45.40	1.17	45.40	1.17
PNIPAAm_164_-b-PLL_45_	53.5	7.50	11.80	44.60	1.21	43.50	1.16
PNIPAAm_164_-b-PLL_113_	73.1	21.90	29.60	53.80	1.27	52.70	1.18
PNIPAAm_102_-PAAm_26_-b-PLL_90_	85.7	21.40	23.60	39.30	1.24	40.20	1.13
PNIPAAm_68_-PAAm_34_-b-PLL_73_	77.8	19.40	19.15	34.40	1.27	36.80	1.20
PNIPAAm_129_-PAAm_39_-b-PLL_82_	70.65	17.65	21.50	47.00	1.24	45.70	1.21
PNIPAAm_219_-PAAm_58_-b-PLL_88_	90	23.40	23.00	53.90	1.32	50.40	1.22

### 2.5. Lower Critical Solution Temperature

Certain water soluble polymers, like PNIPAAm, exhibit a lower critical solution temperature (LCST). This temperature is dependent on the concentration of the aqueous solution [50,51], the polarity of end groups [52], the size of the polymer chains [51,52,53] and the presence of salts in water [54]. 

Turbidity measurements were performed on PNIPAAm samples with different end groups to investigate the influence of these end groups on the LCST (Table 4). The cloud point of PNIPAAm (50:1:2:2) was increased by 6.1 °C when the phthalimide end group was removed and the amine group was present. In the case of PNIPAAm with a higher molecular weight (100:1:2:2), the effect of removing the hydrophobic phthalimide group was less pronounced and an increase of 3 °C was observed. 

The effect of the molecular weight on the LCST was also clear. A difference of ~2–6 °C between the PNIPAAm (50:1:2:2) and PNIPAAm (100:1:2:2) for different end groups was discovered (Table 4).

**Table 4:** Influence of end groups on the cloud point of the PNIPAAm macroinitiators. Measurements were carried out with a 3 mg/mL solution.

Sample	Cloud point, °C	
	Protected	Deprotected
PNIPAAm (50:1:2:2)	39.2	45.3
PNIPAAm (100:1:2:2)	36.9	39.9

The LCST of the diblock copolymers proved difficult to measure via turbidity. At low concentrations (0.5–1.5 mg/mL), no visible transition was observed. At high concentrations (1.5–3.0 mg/mL), an ambiguous transition occurred and sedimentation of the diblock copolymer became problematic during the analysis. The lack of a visible transition, at low concentrations, is ascribed to a combination of insensitive measuring equipment and the formation of micelles. 

**Figure 5:**
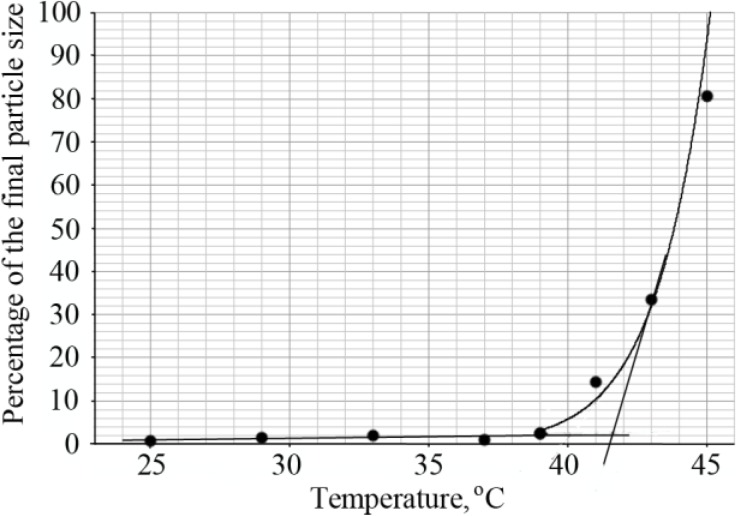
Determination of the LCST of the diblock copolymer PNIPAAm_58_-b-PLL_44_ by DLS.

The LCST of diblock copolymers was determined by dynamic light scattering. The hydrodynamic radius of the copolymer chains was calculated from the diffusion coefficient using the Stokes-Einstein equation, in the temperature range of 25–45 °C. Upon increasing the temperature, large monodisperse particles were formed and this was considered the transition point. The percentage of these big particles was determined and plotted as a function of temperature as shown in Figure 5. The intersection of lines drawn through the baseline and through the leading edge of the curve determined the transition temperature (Figure 5). 

The LCST of all diblock copolymers was found above 37 °C as demonstrated in Table 5. The influence of the sizes of both blocks on the LCST was investigated. When the degree of polymerization of the PLL block was increased from ~50 to ~100, the LCST increased approximately 0.5–1.5 °C. A similar increase in the degree of polymerization of the PNIPAAm blocks caused a shift of the LCST towards lower temperatures for 2.5–3.5 °C. This effect was more pronounced in diblock copolymers with shorter PLL blocks.

**Table 5:** Results from DLS measurements on synthesized PNIPAAm-b-PLL diblock copolymers. Measurements were carried out with a 0.5 mg/mL solution.

PLL blocks	LCST, °C		
	PNIPAAm block length		
	58	118	164
PLL_~50_	41.5	39.0	38.5
PLL_~100_	43.0	39.5	39.0

In addition, the influence of the hydrophilic AAm-comonomer in PNIPAAm-PAAm chains on the LCST was investigated. DLS measurements showed that 20 mol% of AAm in the PNIPAAm chain caused an increase of the LCST of ~10 °C. The increase in LCST of ~15 °C was observed when turbidity measurements were performed. 

**Figure 6:**
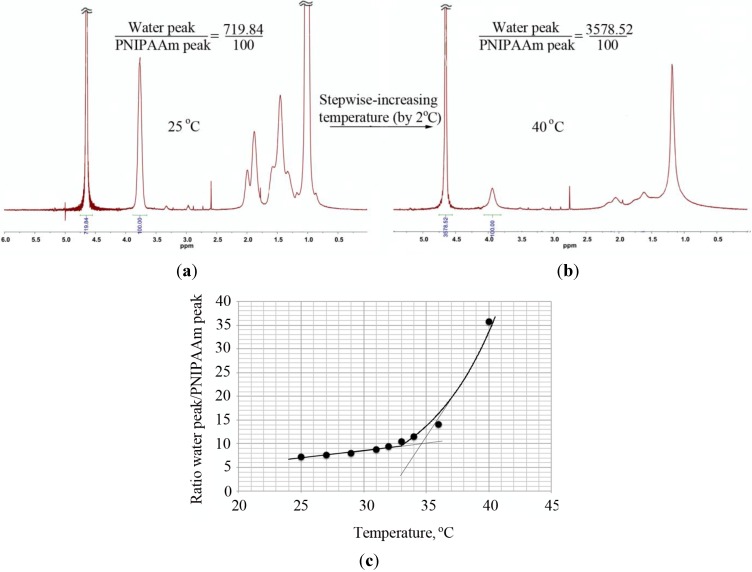
Determination of the LCST of PNIPAAm_58_ by ^1^H-NMR. The ratio of the water peak and the PNIPAAm peak at ~3.9 ppm was determined at measured temperature range (25–40 °C with increments of 2 °C) and plotted as a function of temperature. The intersection of a line drawn through the baseline and through the leading edge of the curve determined the transition temperature. ^1^H-NMR of PNIPAAm at (**a**) 25 °C and (**b**) 40 °C and (**c**) the ratio of the water peak and the PNIPAAm peak plotted as a function of temperature

Besides turbidity and DLS, the transition from a dissolved coil to a collapsed globule was observed by ^1^H-NMR through the changes in the ratio between the water peak and the PNIPAAm peak at 3.9 ppm with an increase of temperature (Figure 6). In the case of the PNIPAAm homopolymers, the LCST obtained in this way was similar to the value determined by DLS and lower than the LCST obtained via turbidity measurements. Contrary to these observations, a large discrepancy in the LCST values (DLS and ^1^H-NMR) of PNIPAAm-PAAm copolymer was obtained (Table 6). Due to lack of time, ^1^H-NMR measurements were performed only once. In order to validate the LCST values obtained via ^1^H-NMR measurements, further experiments will be required.

**Table 6:** LCST of PNIPAAm (Table 1, Ratio 100:1:2:2) and PNIPAAm-PAAm (entry 4, Table 2) determined by turbidity, DLS and ^1^H-NMR.

Sample	Turbidity, °C	DLS, °C	^1^H-NMR, °C
PNIPAAm	39.9	33	34.5
PNIPAAm-PAAm	55	42.5	56

## 3. Discussion

PNIPAAm is a thermo-responsive polymer. Below 32 °C, PNIPAAm is soluble in water. When an aqueous solution of this polymer is heated above this temperature, a transition from dissolved coil into the collapsed globule occurs. Despite the increased hydrophobicity above the LCST, PNIPAAm polymer brushes have shown to provide effective protection from protein adsorption not only below but also above LCST [5,6,7,14,15]. This feature of PNIPAAm brushes provides the applicability of these polymers on alginate beads surface with the aim to provide anti-biofouling properties. Since PNIPAAm cannot be synthesized from the alginate surface directly, the binding with alginate should be accomplished through electrostatic complex formation with the ammonium groups of PLL. Therefore, a PNIPAAm-b-PLL diblock copolymer was synthetized by combining ATRP and ROP as described in Scheme I.

Atom transfer radical polymerization is one of the most frequently used polymerization methods which provides good control over molecular weight and polydispersity. Initially, ATRP was considered unsuitable for the controlled synthesis of polyacrylamides because disproportionation of the copper catalyst, dissociation of the halide ligand, complex formation with (poly)acrylamides and/or solvent as a ligand and disproportionation or hydrolysis of the initiator/dormant chain end and nucleophilic displacement of the terminal halogen atom by the amide group readily take place [55]. Later studies suggested that with a careful choice of solvent, ligand and initiator, these side reactions can be suppressed, and narrowly dispersed polymer can be obtained [30,32,53].

Some research groups have shown that the strong ligand Me_6_TREN allows for the successful ATRP of acrylamides under mild conditions [31,55,56]. Therefore, this ligand was synthetized and used in combination with Cu(I)Cl, the phthalimide initiator and DMSO as a solvent in the ATRP of NIPAAm. In order to avoid premature termination caused by the presence of oxygen, degassing via several freeze-pump-thaw cycles was performed. After applying this method, the achieved conversions were consistent and high (~80%). The obtained polymers were characterized by GPC and ^1^H-NMR (Table 1). The molecular weights of PNIPAAm’s were estimated via end-group analysis with ^1^H-NMR. The molecular weights obtained from ^1^H-NMR analysis were similar to the molecular weights calculated from the initial monomer/initiator ratio and conversion (theoretical molecular weight). On the other hand, there was a consistently high difference between the theoretical molecular weights and the molecular weights determined by GPC. The difference between found and theoretical molecular weight was >100% using universal calibration. When light scattering was used as a detector, the difference was lower (50%–100%).

The disagreement between expected molecular weights and molecular weights determined by either universal calibration or light scattering has been reported in literature [28,52,53]. Xia *et al.* [53] found that molecular weights obtained by GPC were twice as high as the molecular weights determined from ^1^H-NMR (end group analysis) or Maldi-TOF. This difference in values was attributed to a deterioration of the GPC columns in the intervening time. They only used GPC to reveal trends in molecular weights rather than to precisely determine the molecular weights of PNIPAAm [52,53]. 

The LCST of PNIPAAm can be shifted towards higher temperatures by incorporating hydrophilic acrylamide units. Random copolymers of NIPAAm and AAm were prepared by ATRP. When 20 mol% of the NIPAAm monomer units in the starting reaction mixture was replaced by AAm, a significant decrease in conversion was observed. We assume that (P)AAm forms a complex with copper, which as a consequence leads to the deactivation of the catalyst. In order to suppress this side reaction, different polymerization conditions were examined. Among all used solvent systems, DMF provided the best control over the polydispersity, and conversions of ~40% were achieved. Somewhat higher conversions (up to 50%) were achieved when the initial monomer concentration was increased from 33% to 50%.

After successful deprotection of the phthalimide end-groups, confirmed by FTIR and ^1^H-NMR as shown in Figure 2, amino functionalized PNIPAAm homopolymer and PNIPAAm-PAAm copolymer were used as macroinitiators for the ROP of L-lysine NCA. The successful diblock copolymer formation was confirmed by the shift to higher molecular weight in the GPC chromatogram of diblock copolymer compared with the corresponding macroinitiator. Huang *et al.* [33] reported bimodal GPC chromatograms and a PDI > 1.4 when primary amine end-functionalized PNIPAAm was used as an initiator in the ROP of NCAs. The loss of control was attributed to the activated monomer mechanism which coexists with the normal amine mechanism. This effect was eliminated by replacement of the primary amine functionality with amine hydrochloride [33]. Contrary to these findings, the PNIPAAm-PLL and PNIPAAm-PAAm-PLL copolymers described in this paper had a low polydispersity, and no bimodal GPC chromatograms (Figure 3) were observed despite the use of amino-functionalized macroinitiators. The theoretical molecular weights of the PLL block were in good agreement with the values obtained from ^1^H-NMR analysis.

The influence of the molecular weight and end-group on the LCST of PNIPAAm was investigated. The increase in molecular weight caused a decrease in the LCST as demonstrated in Table 4. This effect is attributed to the reduced entropy of mixing with the increase in the molecular weight [53]. Removal of hydrophobic phthalimide shifted the LCST towards higher temperatures due to formation of the hydrophilic amino group on the polymer chain-end. This effect was less remarkable for longer chains. With an increasing degree of polymerization, the contribution of end-group to the hydrophobic/hydrophilic balance of the polymer was reduced. 

After successful removal of the Cbz-group, the temperature dependent behaviour of the diblock copolymers was examined by DLS. The increase in the size of hydrophilic PLL block shifts the LCST towards higher temperatures whereas the length of PNIPAAm block has an opposite effect. As the size of the PLL block was increased, the LCST was shifted to higher temperatures and the phase separation of the diblock copolymer became less apparent.

The presence of 20 mol% of AAm in PNIPAAm chain caused an increase in LCST of approximately 10 °C. This result is in a good agreement with the values reported in literature [20,46,47].

Diversity in LCST values determined by different methods (turbidity, DLS and ^1^H-NMR) is attributed to the sensitivity of the detectors, as well as the polymer concentration in water [50,51] (0.5 mg/mL in DLS, 3 mg/mL in turbidity and 20 mg/mL in ^1^H-NMR). 

## 4. Materials and Methods

### 4.1. Materials

All materials were used as received unless mentioned otherwise.

Ethanolamine (99%, Sigma Aldrich, Zwijndrecht, The Netherlands), di-tert-butyl dicarbonate (99%, Sigma-Aldrich), triethylamine (Sigma-Aldrich), 2-bromoisobutyryl bromide (Sigma-Aldrich), ethylenediamine (≥99%, Fluka, Sigma-Aldrich), (Merck, Amsterdam, The Netherlands), phthalimide (99%, Sigma Aldrich) diethylazodicarboxylic acid (40% in toluene, Fluka, Zwijndrecht, The Netherlands), Silica gel (Type 9385, mesh 230–400, 60 Å, Merck), hydrazine monohydrate (approx. 64%, Sigma-Aldrich), Cu(I)Cl (stored under argon atmosphere, Aldrich, Zwijndrecht, The Netherlands), acrylamide (for electrophoresis, ≥99% (HPLC), Sigma-Aldrich), aluminium oxide Al_2_O_3_ (basic 90, Merck), α-pinene (Sigma-Aldrich), Cbz-L-lysine (Sigma-Aldrich) and triphosgene (Sigma-Aldrich) were used as received. Me_6_TREN was synthesized using formaldehyde (37 wt% in H_2_O, Sigma-Aldrich), formic acid (analysis grade, stored at 6 °C, Acros Organics, Geel, Belgium) and tris (2-aminoethyl)amine (96%, Acros Organics) according to a slightly modified procedure previously described elsewhere [42,57]. Before use *N*-isopropylacrylamide (99% pure, stabilized, Acros Organics) was recrystallized twice from hexane.

### 4.2. Characterization

^1^H-NMR was conducted on a Varian VXR 400 (400 MHz) at room temperature. ^1^H-NMR was also used in the thermal characterization of the copolymers. Copolymers were dissolved at a concentration of ~20 mg/mL in D_2_O. The samples were heated to the measuring temperature and left 30 min to reach thermal equilibrium. ^1^H-NMR spectra were recorded with 100 s relaxation delay and 32 scans. 

Gel Permeation Chromatography (GPC) measurements were performed in a 0.05 M LiBr DMF solution at 50 °C on a Viscotek VE2001 (Viscotek Corporation-A Malvern Company, Houston, TX, USA) with a GRAM 10 Micro Max column (purchased from Polymer Standards Service, Silver Spring, MD, USA) and calibrated with PMMA standards (Agilent Technologies, Santa Clara, CA, USA). Detection was carried out by the Viscotek TDA 302. 

Differential Scanning Calorimetry (DSC) measurements were performed on a Q1000 DSC (TA Instruments, New Castle, DE, USA). DSC was used to accurately determine the melting point of the lysine monomer. The measurements were performed by heating and cooling at a rate of 10 °C/min.

Elemental analysis was performed on Inductively Coupled Plasma-Optical Emission Spectroscopy (ICP-OES) (Perking-Elmer Optima 7000 DV, PerkinElmer Inc., Waltham, MA, USA). Prior to measuring, the samples were destroyed by dissolution in aqua regis (1:3 nitric acid: hydrochloric acid v/v) upon heating to 80 °C. An aliquot of aqua regis served as a blank reference and was subtracted from the measured value. 

Turbidity measurements were performed on a Lauda RC 6 CP (Lauda-Königshofen, Germany). The sample was dissolved in demineralized water at a concentration of 3 mg/mL unless mentioned otherwise. The dissolved sample was placed in the glass cuvette. The distance of the cuvette used for the measurement was 1 cm. The turbidity was measured in arbitrary units. Transmittance was recorded at 0° and light scattering was detected at 90°. The transmittance and light scattering signals were used to calculate the onset of turbidity therefore LCST. Measurements were carried out by heating and cooling with 1 °C/min unless mentioned otherwise. The LCST was defined as the intersection of the baseline and the leading edge of the curve.

DLS measurements were performed on an ALV-CGS-3 goniometer (ALV-GmbH, Langen, Germany) equipped with a LSE-5005 multiple τ digital correlator. Angles between 30° and 130° with a 20° interval were used to measure the decay time twice. These values were averaged and accordingly used to calculate the diffusion coefficient. The hydrodynamic volume was calculated via the Stokes-Einstein equation. The samples were dissolved in demineralized water (0.5 mg/mL) and subsequently filtered with a 0.45 μm cellulose-acetate syringe filter (Whatman, FP30/0.45 CA-s, Eindhoven, The Netherlands). DLS measurements were started at room temperature with a temperature increase of 1–2 °C until a clear transition was observed. After a transition was observed, the diameter of the phase-separated particles was used as a reference. On all temperatures, the relative abundance of the particles with a final diameter was determined and used to follow the transition.

Infrared measurements were performed on a Bruker IFS 88 (Bruker, Bremen, Germany). Samples were dissolved in THF, solvent cast on a silica wafer and measured in transmission.

### 4.3. Synthesis of Di-Functional Phthalimide-Protected Initiator

The phthalimide end capped initiator was synthesized according to the previously described two step procedure [33]. In the first reaction step, ethanolamine was reacted with 2-bromoisobutyryl bromide to yield 2-bromo-*N*-(2-hydroxyethyl)-2-methylpropionamide. This compound served as a starting material in the subsequent synthesis step where the final product phthalimidoethyl 2-bromo-2-methylpropionamide was obtained.

#### 4.3.1. Step 1: 2-Bromo-*N*-(2-Hydroxyethyl)-2-Methylpropionamide

Firstly, 2.66 g (43.5 mmol) of ethanolamine and 4.4 g (87 mmol) of triethylamine were dissolved in 200 mL of dry THF under argon atmosphere. The resulting solution was cooled to 0°C in an ice bath and 10 g (43.5 mmol) of 2-bromoisobutyryl bromide was added dropwise to the mixture. After reacting overnight at room temperature under argon atmosphere, a white precipitate was formed (triethylammonium bromide). This precipitate was filtered off and the solvent was removed by rotary evaporation. The product was purified by silica gel column chromatography (hexane/ethyl acetate v/v: 1/1). The progress of the silica gel column was followed by means of thin layer chromatography (TLC). Yield: 45%, ^1^H-NMR (Varian VXR, 400 MHz, CDCl_3_), δ = 1.95 ppm (s, CBr(CH_3_)_2_), δ = 2.60 ppm (s, CH_2_–OH), δ = 3.43 ppm (q, NH–CH_2_–CH_2_), δ = 3.75 ppm (t, CH_2_–CH_2_–OH), δ = 7.15 ppm (s, CONHCH_2_).

#### 4.3.2. Step 2: Phthalimidoethyl 2-Bromo-2-Methylpropionamide

In the second synthesis step, 3.30 g (15.7 mmol) of intermediate product, 5.35 g (20.4 mmol) of triphenylphosphine (TTP) and 2.31 g (15.7 mmol) of phthalimide were dissolved in approximately 90 mL of dry THF. 8.91 g (20.4 mmol) of 40 wt% toluene solution of diethylazodicarboxylic acid (DEAD) was added dropwise to the reaction mixture. The reaction mixture was left to stir for 12 h at room temperature under an argon atmosphere. The solvents were removed by rotary evaporation and the final product was purified by silica gel column (hexane/ethyl acetate v/v: 4/1) and recrystallized with THF/hexane to yield white needle like crystals. Progress of the silica gel column was followed by means of TLC. Yield: 51%, ^1^H-NMR (Varian VXR, 400 MHz, CDCl_3_), δ = 1.89 ppm (s, CBr(CH_3_)_2_), δ = 3.58 ppm (q, NH–CH_2_–CH_2_), δ = 3.91 ppm (t, CH_2_–CH_2_–N(CO)_2_), δ = 7.10 ppm (s, CONHCH_2_), δ = 7.74 ppm (m, aromatic ring), δ = 7.85 ppm (m, aromatic ring).

### 4.4. A Typical Polymerization of NIPAAm and AAm

NIPAAm polymerization was performed in a dry Schlenk tube (home-made). A typical polymerization with a 100:1:2:2 (monomer:initiator:ligand:catalyst) ratio was performed as follows. The monomer (2.5 g, 22 mmol) and initiator (0.075 g, 0.2 mmol) were placed in a dry Schlenk tube and dissolved in dry DMSO. The ligand (Me_6_TREN, 0.102 g, 0.44 mmol) was dissolved separately in the same solvent. In order to remove all oxygen, both solutions were subjected to five freeze-pump-thaw cycles before use. Catalyst (Cu(I)Cl, 0.044 g, 0.44 mmol) was weighed in a 3-necked flask and the atmosphere was replaced with argon. After the freeze-pump-thaw cycles, the ligand solution was transferred to the flask with catalyst and the obtained solution was allowed to complex for 45–60 min before transferring to the monomer/initiator solution using an argon-flushed syringe. All transfers were done under argon overpressure. All polymerizations were carried out at room temperature unless mentioned otherwise.

After stirring for 24 h, the final conversion was determined as described in the Supplementary data, and the reaction mixture was concentrated and precipitated in a 20-fold excess of ether. The obtained polymer was filtrated and redissolved in THF. In order to remove the catalyst complex, Dowex exchange resin was added to the polymer solution in a polymer:resin = 2.5:1 ratio (wt/wt) and stirred overnight after which the resin beads were removed by filtration. The polymer solution was passed through an Al_2_O_3_ column, concentrated via rotary evaporation and precipitated in a 20-fold excess of ether. The removal of the copper-ligand complex was verified by elemental analysis via ICP-OES.

A random copolymer of NIPAAm and AAm was synthesized by ATRP using the same initiator. The influence of the solvent system, NIPAAm/AAm ratio, initiator/catalyst complex ratio and temperature on the conversion, molecular weight and polydispersity was investigated. The conversion of both NIPAAm and AAm monomers was calculated as described in the Supplementary data. 

The purification of the random copolymer required a higher amount of Dowex exchange resin (polymer:resin= 1:1 ratio). Isolation of the random copolymer, deprotection and copolymerization were performed under the same conditions as described for PNIPAAm homopolymer.

### 4.5. Deprotection of the Phthalimide Protecting Group

The phthalimide protecting group was removed according to literature procedures [33]. PNIPAAm (0.5 g) was dissolved in 2.5 mL of absolute ethanol under an argon atmosphere. A five-fold molar excess of hydrazine monohydrate was added to this solution via a syringe through a rubber septum. Upon adding the hydrazine monohydrate, the solution turned pale yellow due to the formation of phthalyl hydrazide. The resulting solution was left to react for 3 days under an argon atmosphere. Subsequently, dialysis was performed over 2 days (MWCO = 3500, Spectro/Por) at 5 °C. Water was exchanged frequently. The polymer was isolated via lyophilisation and the removal of the phthalimide group was confirmed via ^1^H-NMR by the absence of the aromatic protons of phthalimide group (δ = 7.72 ppm and δ = 7.85 ppm). Yield: 75%.

### 4.6. Synthesis of NCA Lysine

The NCA synthesis of carbobenzyloxy protected lysine was performed based on a previously described procedure [46]. *N*_6_-carbobenzyloxy-L-lysine (11.21 g, 40 mmol) was dissolved in 100 mL of dry THF under an inert argon atmosphere. Subsequently, 20 g (80 mmol) of α-pinene was added and the reaction mixture was heated to 50 °C. After 30 min, 5.38 g (18 mmol) of triphosgene was added and the reaction mixture was stirred for another 2–3 h at 50 °C. When a clear light yellow solution was formed, the reaction mixture was transferred to a double Schlenk tube with a filter, filtered and concentrated to approximately half of the initial reaction volume. The product was purified by multiple recrystallization steps from THF solution into hexane. Residues of hexane and THF were removed under reduced pressure and the product was stored under argon atmosphere at −20 °C. DSC analysis of the melting point was used to give an indication of the purity of the product (m.p. ~100 °C). Yield: 82%. ^1^H-NMR (Varian VX 400, CDCl_3_), δ = 3.21 (m, NHCH_2_), δ = 4.27 (t, CH_2_NHCHCO), δ = 5.11 (s, C_6_H_5_CH_2_O), δ = 7.36 (m, C_6_H_5_). 

### 4.7. Ring Opening Polymerization of NCA Lysine

The polymerization of NCA lysine was performed as follows. Both NCA lysine and macroinitiator (amino-terminated PNIPAAm/PNIPAAm-PAAm) were dried under high vacuum (0.2 mbar) for 1 h at room temperature. The monomer and macroinitiator were dissolved in dry DMF under argon overpressure and a portion of the macroinitiator solution was transferred to the monomer solution via an argon flushed syringe to obtain a 9 wt% solution [33]. The reaction mixture was stirred for 5 days at room temperature under inert argon atmosphere and was then exposed to the air, concentrated and precipitated in a 20-fold excess of diethyl ether. The remaining solid powder was filtered off and left to dry in a vacuum oven at 50 °C.

The degree of polymerization and subsequently molecular weight of the PLL block was determined from ^1^H-NMR as described in the Supplementary data.

### 4.8. Deprotection of the Carbobenzyloxy Protecting Group

The carbobenzyloxy (Cbz) protecting group was removed by dissolving 0.25 g of the diblock copolymer in 7.5 mL of glacial acetic acid. When 1.9 mL of HBr (33 wt% in acetic acid) was added dropwise, the reaction mixture turned into slurry. After 1 h, the reaction mixture was precipitated in a 10-fold excess of diethyether. The precipitate was filtrated, washed with ether and subsequently dissolved in demineralized water. The pH was adjusted to ~6–7 with saturated sodium bicarbonate solution. The diblock copolymer was further purified by dialysis for 2 days (MWCO = 3500). The water for dialysis was exchanged on a regular basis. The deprotected polymer was isolated via lyophilization. ^1^H-NMR of the final polymer confirmed the absence of peaks at δ = 4.95 (s, C_6_H_5_CH_2_O) and δ = 7.24 (m, C_6_H_5_). Yield: 75%.

## 5. Conclusions

Diblock copolymers of PNIPAAm or PNIPAAm-PAAm and PLL were successfully synthetized by combining ATRP and ROP. The PNIPAAm homopolymer and PNIPAAm-PAAm random copolymer with low dispersity were prepared by ATRP in the presence of a strong ligand (Me_6_TREN), Cu(I)Cl and the phthalimide di-functional initiator. Lower conversions were observed when AAm monomers were present in the reaction mixture (random copolymerization of NIPAAm and AAm) than when NIPAAm was polymerized alone. The obtained polymers were characterized by ^1^H-NMR and GPC. The molecular weights determined by GPC were >1.5 times higher than the molecular weights obtained from the end-group analysis in ^1^H-NMR. Amino-terminated PNIPAAm homopolymers and PNIPAAm-PAAm copolymers were used as macroinitiators in the ROP of lysine NCA. Successful copolymer formation was confirmed by the shift to higher molecular weights in the GPC chromatogram of diblock copolymers (PNIPAAm-b-PLL or PNIPAAm-PAAm-b-PLL) compared to the corresponding macroinitiators (PNIPAAm or PNIPAAm-PAAm). The thermal behavior of the prepared homopolymers and copolymers was examined by DLS, turbidity and ^1^H-NMR. It was shown that the length of PNIPAAm, the nature of polymer-end group and the copolymerization with other hydrophilic monomers all influence the LCST.

## Supplementary Materials

Supplementary File 1

## References

[1] Franzin C.M., Macdonald P.M., Polozova A., Winnik F.M. (1998). Destabilization of cationic lipid vesicles by an anionic hydrophobically modified poly(*N*-isopropylacrylamide) copolymer: A solid-state 31P NMR and 2H NMR study. Biochim. Biophys. Acta.

[2] Qin S., Geng Y., Discher D.E., Yang S. (2006). Temperature-controlled assembly and release from polymer vesicles of poly(ethylene oxide)-block-poly(*N*-isopropylacrylamide). Adv. Mater..

[3] Chu L.-Y., Park S.-H., Yamaguchi T., Nakao S.-I. (2001). Preparation of thermo-responsive core-shell microcapsules with a porous membrane and poly(*N*-isopropylacrylamide) gates. J. Membr. Sci..

[4] Choi S., Choi B.-C., Xue C., Leckband D. (2012). Protein adsorption mechanisms determine the efficiency of thermally controlled cell adhesion on poly(*N*-isopropyl acrylamide) brushes. Biomacromolecules.

[5] Fukumori K., Akiyama Y., Kumashiro Y., Kobayashi J., Yamato M., Sakai K., Okano T. (2010). Characterization of ultra-thin temperature-responsive polymer layer and its polymer thickness dependency on cell attachment/detachment properties. Macromol. Biosci..

[6] Akiyama Y., Kikuchi A., Yamato M., Okano T. (2004). Ultrathin poly(*N*-isopropylacrylamide) grafted layer on polystyrene surfaces for cell adhesion/detachment control. Langmuir.

[7] Fukumori K., Akiyama Y., Yamato M., Kobayashi J., Sakai K., Okano T. (2009). Temperature-responsive glass coverslips with an ultrathin poly(*N*-isopropylacrylamide) layer. Acta Biomater..

[8] Kanazawa H., Yamamoto K., Matsushima Y., Takai N., Kikuchi A., Sakurai Y., Okano T. (1996). Temperature-responsive chromatography using poly(*N*-isopropylacrylamide)-modified silica. Anal. Chem..

[9] Mizutani A., Nagase K., Kikuchi A., Kanazawa H., Akiyama Y., Kobayashi J., Annaka M., Okano T. (2010). Thermo-responsive polymer brush-grafted porous polystyrene beads for all-aqueous chromatography. J. Chromatogr. A.

[10] Kanazawa H., Okano T. (2011). Temperature-responsive chromatography for the separation of biomolecules. J. Chromatogr. A.

[11] Yang J., Yamato M., Kohno C., Nishimoto A., Sekine H., Fukai F., Okano T. (2005). Cell sheet engineering: Recreating tissues without biodegradable scaffolds. Biomaterials.

[12] Nagase K., Kobayashi J., Okano T. (2009). Temperature-responsive intelligent interfaces for biomolecular separation and cell sheet engineering. J. R. Soc. Interface.

[13] Takahashi H., Nakayama M., Yamato M., Okano T. (2010). Controlled chain length and graft density of thermoresponsive polymer brushes for optimizing cell sheet harvest. Biomacromolecules.

[14] Burkert S., Bittrich E., Kuntzsch M., Müller M., Eichhorn K.-J., Bellmann C., Uhlmann P., Stamm M. (2009). Protein resistance of PNIPAAm brushes: Application to switchable protein adsorption. Langmuir.

[15] Xue C., Yonet-Tanyeri N., Brouette N., Sferrazza M., Braun P.V., Leckband D.E. (2011). Protein adsorption on poly(*N*-isopropylacrylamide) brushes: Dependence on grafting density and chain collapse. Langmuir.

[16] Schild H. (1992). Poly(*N*-isopropylacrylamide): Experiment, theory and application. Prog. Polym. Sci..

[17] Dimitrov I., Trzebicka B., Müller A.H.E., Dworak A., Tsvetanov C.B. (2007). Thermosensitive water-soluble copolymers with doubly responsive reversibly interacting entities. Prog. Polym. Sci..

[18] Taylor L.D., Cerankowski L.D. (1975). Preparation of films exhibiting a balanced temperature dependence to permeation by aqueous solutions—A study of lower consolute behavior. J. Polym. Sci. A Polym. Chem. Ed..

[19] Feil H., Bae Y.H., Feijen J., Kim S.W. (1993). Effect of comonomer hydrophilicity and ionization on the lower critical solution temperature of *N*-isopropylacrylamide copolymers. Macromolecules.

[20] Shen Z., Terao K., Maki Y., Dobashi T., Ma G., Yamamoto T. (2006). Synthesis and phase behavior of aqueous poly(*N*-isopropylacrylamide-co-acrylamide), poly(*N*-isopropylacrylamide-co-*N,N*-dimethylacrylamide) and poly(*N*-isopropylacrylamide-co-2-hydroxyethyl methacrylate). Colloid Polym. Sci..

[21] Schilli C.M., Zhang M., Rizzardo E., Thang S.H., Chong Y.K., Edwards K., Karlsson G., Müller A.H.E. (2004). A new double-responsive block copolymer synthesized via RAFT polymerization:  Poly(*N*-isopropylacrylamide)-block-poly(acrylic acid). Macromolecules.

[22] Savariar E.N., Thayumanavan S. (2004). Controlled polymerization of *N*-isopropylacrylamide with an activated methacrylic ester. J. Polym. Sci. A Polym. Chem..

[23] Ito M., Ishizone T. (2006). Living anionic polymerization of *N*-methoxymethyl-*N*-isopropylacrylamide: Synthesis of well-defined poly(*N*-isopropylacrylamide) having various stereoregularity. J. Polym. Sci. Part. A Polym. Chem..

[24] Aoshima S., Kanaoka S. (2008). Synthesis of stimuli-responsive polymers by living polymerization: Poly(*N*-isopropylacrylamide) and poly(vinyl ether)s. Wax Crystal Control Nanocomposites Stimuli-Responsive Polymers.

[25] Audouin F., Heise A. (2013). Surface-initiated RAFT polymerization of NIPAM from monolithic macroporous polyHIPE. Eur. Polym. J..

[26] Wang X., Li S., Su Y., Huo F., Zhang W. (2013). Aqueous RAFT polymerization of *N*-isopropylacrylamide-mediated with hydrophilic macro-RAFT agent: Homogeneous or heterogeneous polymerization?. J. Polym. Sci. Part A Polym. Chem..

[27] Ganachaud F., Monteiro M.J., Gilbert R.G., Dourges M.-A., Thang S.H., Rizzardo E. (2000). Molecular weight characterization of poly(*N*-isopropylacrylamide) prepared by living free-radical polymerization. Macromolecules.

[28] Schilli C., Lanzendörfer M.G., Müller A.H.E. (2002). Benzyl and cumyl dithiocarbamates as chain transfer agents in the RAFT polymerization of *N*-isopropylacrylamide. *In situ* FT-NIR and MALDI−TOF MS investigation. Macromolecules.

[29] Appel E.A., del Barrio J., Loh X.J., Dyson J., Scherman O.A. (2012). High molecular weight polyacrylamides by atom transfer radical polymerization: Enabling advancements in water-based applications. J. Polym. Sci. Part A Polym. Chem..

[30] Vachaudez M., D’hooge D.R., Socka M., Libiszowski J., Coulembier O., Reyniers M.F., Duda A., Marin G.B., Dubois P. (2013). Inverse dependencies on the polymerization rate in atom transfer radical polymerization of *N*-isopropylacrylamide in aqueous medium. React. Funct. Polym..

[31] Masci G., Giacomelli L., Crescenzi V. (2004). Atom transfer radical polymerization of *N*-isopropylacrylamide. Macromol. Rapid Commun..

[32] Millard P.-E., Mougin Nathalie C., Böker A., Müller Axel H.E. (2009). Controlling the fast ATRP of *N*-isopropylacrylamide in water. Controlled/Living Radical Polymerization: Progress in ATRP.

[33] Huang C.-J., Chang F.-C. (2008). Polypeptide diblock copolymers: Syntheses and properties of poly(*N*-isopropylacrylamide)-b-polylysine. Macromolecules.

[34] Hadjichristidis N., Iatrou H., Pitsikalis M., Sakellariou G. (2009). Synthesis of well-defined polypeptide-based materials via the ring-opening polymerization of α-amino acid *N*-carboxyanhydrides. Chem. Rev..

[35] Kricheldorf H.R. (2006). Polypeptides and 100 years of chemistry of α-amino acid *N*-carboxyanhydrides. Angew Chem. Int. Ed. Engl..

[36] Penczek S., Kricheldorf H.R. (1990). Models of Biopolymers by Ring Opening Polymerization.

[37] Idelson M. (1985). Poly(γ-benzyl-L-glutamate) and other glutamic-acid-containing polymers. J. Polym. Sci. Polym. Lett. Ed..

[38] Deming T.J. (1997). Polypeptide materials: New synthetic methods and applications. Adv. Mater..

[39] Cheng J., Deming T.J. (2012). Synthesis of polypeptides by ring-opening polymerization of alpha-amino acid *N*-carboxyanhydrides. Top. Curr. Chem..

[40] Dimitrov I., Schlaad H. (2003). Synthesis of nearly monodisperse polystyrene-polypeptide block copolymers via polymerisation of *N*-carboxyanhydrides. Chem. Commun..

[41] Dimitrov I., Kukula H., Cölfen H., Schlaad H. (2004). Advances in the synthesis and characterization of polypeptide-based hybrid block copolymers. Macromol. Symp..

[42] Queffelec J., Gaynor S.G., Matyjaszewski K. (2000). Optimization of atom transfer radical polymerization using Cu(I)/tris(2-(dimethylamino)ethyl)amine as a catalyst. Macromolecules.

[43] Meyer D.E., Shin B.C., Kong G.A., Dewhirst M.W., Chilkoti A. (2001). Drug targeting using thermally responsive polymers and local hyperthermia. J. Control. Release.

[44] Russo P.S. (1987). Reversible Polymeric Gels and Related Systems.

[45] Nishiyama N., Kataoka K., Satchi-Fainaro R., Duncan R. (2006). Nanostructured devices based on block copolymer assemblies for drug delivery: Designing structures for enhanced drug function. Polymer Therapeutics II.

[46] Daly W.H., Poché D. (1988). The preparation of *N*-carboxyanhydrides of α-amino acids using bis(trichloromethyl)carbonate. Tetrahedron Lett..

[47] Priest John H., Murray Sheryl L., Nelson R.J., Hoffman Allan S. (1987). Lower critical solution temperatures of aqueous copolymers of *N*-isopropylacrylamide and other *N*-substituted acrylamides. Reversible Polymeric Gels and Related Systems.

[48] Izunobi J.U., Higginbotham C.L. (2010). Microstructure characterization and thermal analysis of hybrid block copolymer α-methoxy-poly(ethylene glycol)-block-poly[ε-(benzyloxycarbonyl)-L-lysine] for biomedical applications. J. Mol. Struct..

[49] Hrkach J.S., Ou J., Lotan N., Langer R. (1995). Synthesis of poly(L-lactic acid-co-L-lysine) graft copolymers. Macromolecules.

[50] Zhou X., Li J., Wu C., Zheng B. (2008). Constructing the Phase diagram of an aqueous solution of poly(*N*-isopropyl acrylamide) by controlled microevaporation in a nanoliter microchamber. Macromol. Rapid Commun..

[51] Afroze F., Nies E., Berghmans H. (2000). Phase transitions in the system poly(*N*-isopropylacrylamide)/water and swelling behaviour of the corresponding networks. J. Mol. Struct..

[52] Xia Y., Burke N.A.D., Stöver H.D.H. (2006). End group effect on the thermal response of narrow-disperse poly(*N*-isopropylacrylamide) prepared by atom transfer radical polymerization. Macromolecules.

[53] Xia Y., Yin X., Burke N.A.D., Stöver H.D.H. (2005). Thermal response of narrow-disperse poly(*N*-isopropylacrylamide) prepared by atom transfer radical polymerization. Macromolecules.

[54] Zhang Y., Furyk S., Bergbreiter D.E., Cremer P.S. (2005). Specific ion effects on the water solubility of macromolecules:  Pnipam and the hofmeister series. J. Am. Chem. Soc..

[55] Teodorescu M., Matyjaszewski K. (2000). Controlled polymerization of (meth)acrylamides by atom transfer radical polymerization. Macromol. Rapid Commun..

[56] Neugebauer D., Matyjaszewski K. (2003). Copolymerization of *N,N*-dimethylacrylamide with n-butyl acrylate via atom transfer radical polymerization. Macromolecules.

[57] Opsteen J.A., van Hest J.C.M. (2007). Modular synthesis of ABC type block copolymers by “click” chemistry. J. Polym. Sci. Part A Polym. Chem..

