# Supplementary material for: Synthesis and Phase Behavior of Poly(N-isopropylacrylamide)-b-Poly(L-Lysine Hydrochloride) and Poly(N-Isopropylacrylamide-co-Acrylamide)-b-Poly(L-Lysine Hydrochloride)
Source: Materials (Basel). 2014 Jul 22;7(7):5305–26. doi: 10.3390/ma7075305 (PMC5455825; doi:10.3390/ma7075305)
Supplement: Supplementary File 1 [file materials-07-05305-s001.pdf]

Supplementary Data

Final Conversion Calculations

The final conversion of NIPAAm was determined after 24 h of polymerization by ^{1}H-NMR by integrating the area of the peak of the double bond in the monomer (δ = 5.35 ppm) and the area of 3.66–3.98 ppm ($(CH_3)_2CHNH$ from both monomer and polymer) using the following equation:

$$\text{Conversion} = 1 - \frac{A_{\delta\,=\,5.35\text{ ppm}}}{A_{\delta\,=\,3.98-3.66\text{ ppm}}} \tag{1}$$

The final conversion of NIPAAm comonomer in random copolymerization of NIPAAM and AAm was determined in the same manner as described for the homopolymerization of NIPAAm. The conversion of AAm monomer was calculated based on the NIPAAm conversion and the ratio between the area corresponding to the NIPAAm double bond (δ = 5.49–5.54 ppm) and the area of the AAm double bond (δ = 5.54–5.58 ppm).

$$\text{Conversion(AAm)} = \text{Conversion(NIPAAm)} \times \frac{A_{\delta\,=\,5.54\,-\,5.58\text{ ppm}}}{A_{\delta\,=\,5.49\,-\,5.54\text{ ppm}}} \tag{2}$$

PNIPAAm-b-PLL and PNIPAAm-PAAm-b-PLL: The Degree of Polymerization Calculations

The degree of polymerization and, subsequently, the molecular weight of the PLL block was determined from ^{1}H-NMR using equation:

$$N(\text{PLL}) = \frac{M_n\ (\text{PLL})}{M(\text{Cbz Lysine })} = \frac{\frac{A_{\delta\,=\,2.9\text{ ppm}}}{2}}{\frac{A_{\delta\,=\,2.25\,-\,0.8\text{ ppm}} - (A_{\delta\,=\,2.9\text{ ppm}} \times 3)}{9}} \times \text{N(PNIPAAm)} \tag{3}$$

With: N(PLL)—Degree of polymerization of PLL;
M_n(PLL)—Molecular weight of PLL block;
M(Cbz Lysine)—Molecular weight of carbobenzyloxy-L-lysine repeating unit;
N(PNIPAAm)—Degree of polymerization of PNIPAAm;
A_δ = 2.9 ppm—Integrated area under the peak of two protons ($CH_2NHCOOCH_2C_6H_5$) of PLL;
A_δ = 2.25–0.8 ppm—Integrated area from δ = 2.25 until δ = 0.8 of nine protons ($CH(CH_3)_2$, $BrCHCH_2$ and $BrCHCH_2$) of PNIPAAm and six protons ($CH–(CH_2)_3–CH_2–NH$) from PLL

In case of a PNIPAAm-PAAm-b-PLL copolymer, the following equation was applied:

$$N(\text{PLL}) = \frac{M_n\ (\text{PLL})}{M(\text{Cbz Lysine })} = \frac{\frac{A_{\delta\,=\,2.9\text{ ppm}}}{2}}{\frac{A_{\delta\,=\,2.25-0.8\text{ ppm}} - 3 \times (\frac{N(\text{PAAm})}{N(\text{PNIPAAm})} + A_{\delta\,=\,2.9\,ppm})}{9}} \times N(\text{PNIPAAm}) \tag{4}$$

N(PAAm)—Degree of polymerization of PAAm;
N(PNIPAAm)—Degree of polymerization of PNIPAAm.
